# Circulating Heat Shock Protein 70 Is a Novel Biomarker for Early Diagnosis of Lung Cancer

**DOI:** 10.1155/2018/6184162

**Published:** 2018-08-29

**Authors:** Tielong Tang, Chao Yang, Ham Ebo Brown, Jing Huang

**Affiliations:** ^1^Department of Urology, Urogenital Diseases Lab, Affiliated Hospital of North Sichuan Medical College, Nanchong 637000, China; ^2^Department of Thoracic Surgery, Chongqing Three Gorges Central Hospital, Wanzhou 404100, China

## Abstract

Heat shock protein 70 (HSP70) was a highly conserved protein which was significantly induced in response to cellular stresses. HSP70 played an important role in the pathogenesis of cancer which stabilized the production of large amount of oncogenic proteins and finally supported growth and survival of tumor. However, there was no report about the diagnosis of circulating HSP70 in lung cancer patients. In this study, a total of 297 participants (lung cancer: 197, healthy control: 100) were enrolled in the detection of circulating HSP70 level in plasma by ELISA assay. The results indicated that circulating HSP70 significantly decreased in lung cancer patients compared to healthy controls (*P* < 0.0001). Receiver operating characteristic (ROC) analysis showed that HSP70 (AUC: 82.2%, SN: 74.1%, SP: 80.0%) had higher diagnosis value than clinical existing biomarkers CEA (AUC: 80.1%, SN: 76.8%, SP: 67.3%) and CA 19-9 (AUC: 63.7%, SN: 64.2%, SP: 54.0%). In the analysis of early lung cancer patients, ROC results also revealed that HSP70 (AUC: 83.8%, SN: 71.2%, SP: 84.0%) have higher sensitivity, specificity, and AUC than CEA (AUC: 73.7%, SN: 73.2%, SP: 69.1%) and CA 19-9 (AUC: 61.5%, SN: 69.4%, SP: 53.4%). In analysis of specific histological classifications, HSP70 showed more valuable in the diagnosis of SCC (AUC: 85.9%, SN: 86.1.9%, SP: 81.0%) than ADC (AUC: 81.0%, SN: 69.1%, SP: 81.0%). Combined analysis of HSP70 and existing biomarker: CEA and CA 19-9 exhibited that HSP70 combined CEA and CA 19-9 showed the highest AUC (0.945, 95% CI, 0.855–1.000). The importance of our results was that we found decreased circulating HSP70, in combination with elevated CEA and CA 19-9, could be utilized in the diagnosis of early (stage I and II) lung cancer.

## 1. Introduction

The heat shock protein (HSP) family was a molecular chaperones group which played an important role in protein folding, preventing protein aggregation and transport of proteins across membranes [1]. These functions enabled HSP to play critical roles in regulation of protein homeostasis and cell survival [2]. In condition of cell stress, including environmental (i.e., elevated temperature, oxidative stress, and heavy metal exposure), biological (i.e., cell proliferation and differentiation), and pathological (i.e., inflammation and tumor growth) stress, the expression of HSPs was highly upregulated [3]. HSP70 was an important member of HSP family which expressed in various subcellular localizations. HSP protein consisted of an N-terminal ATPase domain, where target protein could bind and release driving by ATP exchange and a C-terminal domain containing an EEVD motif for cochaperone binding [4].

HSP70 overexpression was correlated with poor prognosis in a wide range of cancers such as breast, prostate, colon, brain, and lung cancer and was necessary for the survival of tumor cells [5–9]. HSP70 was frequently transported and anchored on plasma membrane of tumor and released into the blood in phenotype of exosomes [10]. In bladder cancer analysis, Margel et al. indicated that increased level of HSP70 in urine could distinguish patients with muscle-invasive bladder cancer from nonmuscle-invasive bladder cancer [11]. Lysine methylation of HSP70 could be used as a potential prognostic factor in metastatic high-grade serous carcinoma [12]. In hepatocellular carcinoma (HCC) study, highly expressed HSP70 was a sensitive marker for the diagnosis of well-differentiated small HCC from high-grade dysplastic nodules [13].

Although studies indicated that increased serum levels of HSP70 were observed in HCC, lung cancer and bladder cancer, these assays only focused either on tissues or on HSP70 in membrane phenotype. There was no report about the role of circulating HSP70 in lung cancer diagnosis and the relationship between level of circulating HSP70 and metastasis. In this study, ELISA method was performed to detect the level of circulating HSP70 in plasma from 197 lung cancer patients and 100 healthy controls. The aim of this study was to confirm the role of circulating HSP70 in lung cancer diagnosis especially in early lung cancer patients.

## 2. Materials and Methods

### 2.1. Patients Enrolled in Study

A total of lung cancer (197) and healthy individuals (100) was enrolled in this study. All patients were from the center of cancer in the Affiliated Hospital of North Sichuan Medical College and Chongqing Three Gorges Central Hospital in 2016 and singed informed consent for this study. Blood samples were collected within two weeks of the first biopsy-proven lung cancer diagnosis and prior to surgical procedure and other therapy methods. All healthy controls were from a routine physical examination in the Center of Physical Examination in the Affiliated Hospital of North Sichuan Medical College and Chongqing Three Gorges Central Hospital. Plasmas are collected in EDTA and anticoagulant-free tubes and processed according to the standard protocols. Anticoagulant whole blood (5 ml) was centrifuged to isolate the plasma (800*g*, 20 min, 4°C). The isolated plasma was then centrifuged again to remove the remaining red blood cells and leukocytes (800*g*, 20 min, 4°C). The collected plasma was transferred to 1.5 ml clean Eppendorf tubes and stored in −80°C.

### 2.2. ELISA for Assay of HSP70 Levels

Levels of HSP70 in large plasma samples validation were measured using an ELISA kit purchased from R&D system (SUV 1663). All plasmas of lung cancer patients and healthy controls as well as kit components were equilibrated at room temperature before detection. The assay diluent was used to solve the standard powder and dilute all plasma samples. All diluted samples and standards were added to appropriate wells and then covered with plate sealers. The plates were then incubated for two hours at room temperature. All wells were aspirated to remove the liquid and washed three times with wash buffer. HSP70 detection antibody diluted by reagent diluents was added to each well, covered with a new adhesive strip and then incubated for another two hours at room temperature. After three washes with buffer, 100 *μ*l of diluted streptavidin-HRP was added to each well and incubated for 20 minutes at room temperature. The plates were washed for three times and color development was achieved by adding 100 *μ*l of substrate solution to each well and incubated for 20 minutes at room temperature in a dark place. The reaction was stopped by adding 50 *μ*l sulfuric acid. The optical density (OD) was measured at 450 nm. The concentrations of HSP70 were calculated according to the formula of standard curve. All tested plasma samples as well as standard samples and blank controls were assayed in duplicate to reduce variation.

### 2.3. Statistical Analysis

Chi-square test in SPSS16.0 was used to analyze the protein levels between lung cancer patients and healthy controls and nonparametric analyses by GraphPad Prism version 5 for Windows. Receive operating characteristic (ROC) curves were plotted to evaluate the sensitivity, specificity, and areas under the curves (AUC) with a 95% confidence interval (CI). The optimum cutoff value for diagnosis was determined by maximizing the specificity and sensitivity. A two-tailed *P* value less than 0.05 was considered significant. To assess whether the diagnostic efficiency of HSP70 in combination with carcinoembryonic antigen (CEA) and carbohydrate antigen (CA 19-9) was superior to that of the individual biomarkers alone, new variable models for NSCLCs were created on the basis of the equations obtained by binary logistic regression.

## 3. Results

### 3.1. The Basic Characteristic of Lung Cancer Patients and Controls

There were a total of 297 samples enrolled in the trial including 197 lung cancer patients and 100 healthy controls. NSCLC was defined on the basis of CT according to the World Health Organization Classification of Tumors of the Lung [14]. Tumor stage was defined according to the 7th IASLC/AJCC staging system [15]. The clinical characteristics of lung cancer and healthy controls in large sample verification were shown in Table 1.

### 3.2. The Role of HSP70 as Novel Biomarkers in Lung Cancer Diagnosis

**Figure 1:**
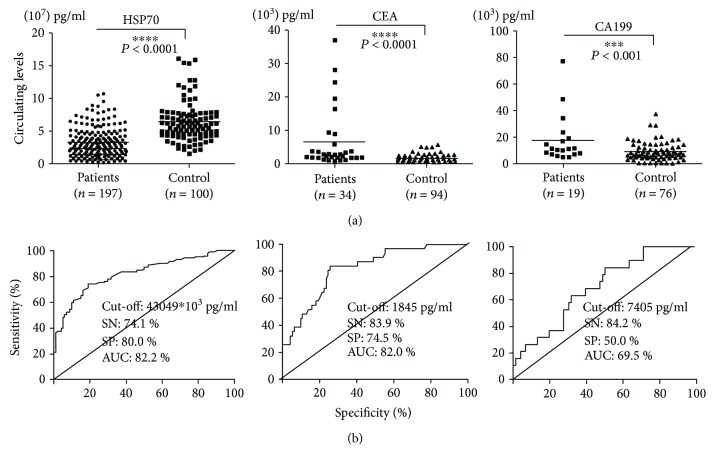
The concentrations and ROC curve analyses of HSP70, CEA, and CA 19-9 in all lung cancer patients. (a) Concentrations; (b) ROC curves. The black horizontal lines are median values. Controls: healthy volunteers in routine physical examination. *P* values were determined by the chi-square test (^∗∗∗^*P* < 0.001, ^∗∗∗∗^*P* < 0.0001).

ELISA assay was conducted to measure free HSP70 levels in plasmas of large cohort samples (197 lung cancer patients and 100 healthy controls) with clear and sufficient clinical records. In the lung cancer patients, the plasma HSP70 levels were dramatically decreased compared with healthy controls (*P* < 0.0001) (Figure 1(a)). In CEA and CA 19-9 analysis, it was shown that these two molecules were significantly upregulated in lung cancer patients (Figure 1(a)).

Receive operating characteristic (ROC) analysis was used to define the sensitivity (SN) and specificity (SP) of HSP70. The results indicated that HSP70 displayed a relatively high SN/SP and AUC (AUC: 82.2%, SN: 74.1%, SP: 80.0%). Furthermore, to compare the diagnostic role of the new biomarker, we also analyzed 2 existing clinical biomarkers, CEA and CA 19-9, in lung cancer and healthy samples and compared their diagnostic efficacy with HSP70. The results indicated that AUC were all less than HSP70 (CEA: AUC: 82.0%, SN: 83.9%, SP: 74.5%; CA 19-9: AUC: 69.5%, SN: 84.2%, SP: 50.0%) (Figure 1(b)). The analysis shows that HSP70 were more sensitive and specific than CEA and CA 19-9, which confirmed the role of HSP70 in lung cancer diagnosis.

### 3.3. The Role of HSP70 in ADC and SCC Patients' Diagnosis

**Figure 2:**
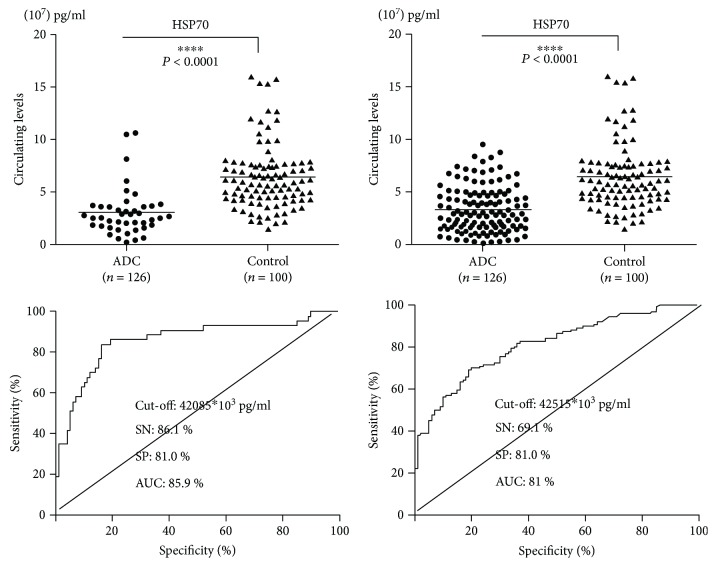
The concentrations and ROC curve analyses of HSP70 in ADC and SCC patients. Controls: healthy volunteers in routine physical examination (^∗∗∗∗^*P* < 0.0001).

In this study, we analyzed the role of HSP70 in adenocarcinoma (ADC) and squamous carcinoma (SCC) patients' diagnosis; the results indicated that plasma HSP70 levels of ADC and SCC were highly significant lower than that of the healthy controls (*P* < 0.0001) (Figure 2). Receive operating characteristic (ROC) analysis indicated that HSP70 had more diagnostic valuable in SCC patients (AUC: 85.9%, SN: 86.1%, SP: 81.0%) than in ADC patients (AUC: 81.0%, SN: 69.1%, SP: 80.0%) (Figure 2). The results revealed that HSP70 had a high diagnostic efficiency in SCC than ADC.

**Figure 3:**
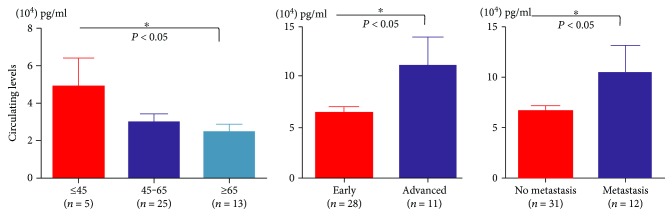
The association analysis between HSP70 levels and age, stages, and metastasis in SCC patients (^∗^*P* < 0.05).

Next, we analyzed the role of HSP70 in pathological characteristic of ADC and SCC patients. We found that HSP70 showed no obvious correlation with pathological characteristic of ADC (data not listed here). As the second subtype of NSCLC, squamous carcinoma (SCC) consisted almost 15% in lung cancer patients. Our analysis based on the ELISA results indicated that circulating HSP70 were dramatically decreased in older SCC patients (>65 years compared with <45 years, *P* < 0.05) (Figure 3), the plasma HSP70 levels in SCC patients has decreased with age as a whole. In addition, the plasma HSP70 levels in SCC patients has prominently decreased in early stage SCC compared with advanced lung cancer patients (*P* < 0.05) (Figure 3). The decreased HSP70 levels were also found in SCC patients without metastasis compared with metastasis group (*P* < 0.05) (Figure 3). The results of this analysis mean the important diagnosis role of HSP70 in SCC patients.

### 3.4. The Role of HSP70 in Early Diagnosis of Lung Cancer

**Figure 4:**
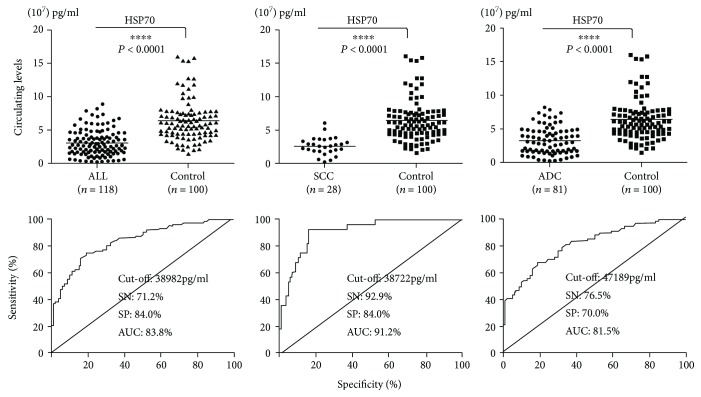
The concentrations and ROC curve analyses of HSP70 in early lung cancer patients. Controls: healthy volunteers in routine physical examination (^∗∗∗∗^*P* < 0.0001).

Early diagnosis of lung cancer always related with higher survival rate (70–80%) than advanced diagnosis patients (15%). To assess the role of HSP70 in detecting early lung cancer, we analyzed the plasma HSP70 levels in early stage lung cancer patients and healthy controls based on the ELISA results. The analysis results suggested that the circulating level of HSP70 in early stage lung cancer patients was significantly lower than that in healthy controls (Figure 4(a)). The plasma HSP70 levels of early stage lung cancer patients with ADCs and SCCs were also both extremely significantly lower than healthy controls (Figure 4(a)).

ROC curves based on the ELISA results were applied to determine the diagnostic efficiency of plasma HSP70 levels for early stage lung cancer patients. The results indicated that HSP70 (AUC: 83.8%, SN: 71.2%, SP: 84.0%) exhibited a well efficacy in all early lung cancer patients (Figure 4(b)). Furthermore, the diagnostic efficiency of plasma HSP70 levels for early lung cancer patients with SCC (AUC: 91.2%, SN: 92.9%; SP: 84.0%) higher than early lung cancer patients than with ADCs (AUC: 81.5%, SN: 76.5%, SP: 70.0%) (Figure 4(b)). Based on observations, we could draw a conclusion that HSP70 has the vital significance to the diagnosis of early lung cancer, especially in SCC. All SN, SP, and AUC were summarized in Table 2.

### 3.5. ROC Analysis of HSP70 and the Construction of Diagnostic Models for Lung Cancer

**Figure 5:**
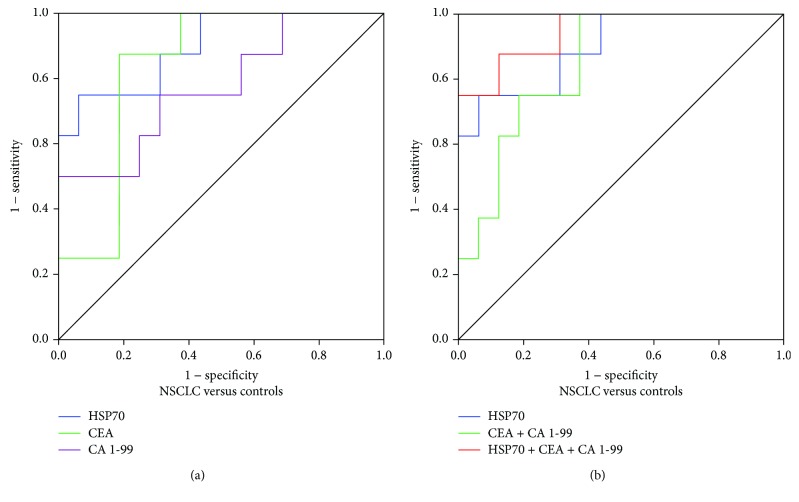
The combination analysis of HSP70, CEA, and CA 19-9. Controls: healthy volunteers in routine physical examination. (a) Individual analysis of HSP70, CEA, and CA 19-9; (b) Comparison of combination of HSP70, CEA, and CA 19-9 with individual HSP70 as well as CEA + CA 19-9.

In this study, receiver operating characteristic (ROC) based on the ELISA results was applied to evaluate the diagnostic efficiency of HSP70 plasma levels for lung cancer. The efficiency of the existing clinical biomarkers CEA and CA 19-9 in distinguishing NSCLCs from healthy controls was also included (Figure 5, Table 2). The measurements of the different individual markers and their predictive value in the diagnosis of NSCLCs are shown in Table 3.

The efficiency of HSP70 in combination with CEA and CA 19-9 was higher than that of the individual biomarkers alone for the discrimination of NSCLCs from healthy controls, showed the highest AUC (0.945, 95% CI, 0.855–1.000). Compared the existing clinical biomarkers CEA and CA 19-9, HSP70 displayed a high AUC (0.898, 95% CI, 0.764-1.000) (Table 3). The results showed that HSP70 exhibited optimal efficacy than that of CEA and CA 19-9 for the diagnosis of lung cancer.

Then, binary logistic regression analyses were used to evaluate the superiority of HSP70 in lung cancer diagnosis; we found that even the combination of CEA and CA 19-9 is not higher than the individual diagnostic efficacy of HSP70 (0.898, 95% CI, 0.764–1.000; CEA + CA 19-9: 0.844, 95% CI, 0.687–1.000).

## 4. Discussion

Recent statistics indicated that lung cancer was still the leading cause of malignancy tumor-related deaths in both the United States and China [16, 17]. The important reason for high mortality of lung cancer was due to late diagnosis since the symptoms (coughing, chest pain, or dyspnea) were quite unspecific [18]. Therefore, earlier detection of lung cancer was a high medical need to increase life expectancy of lung cancer. The most widely used method for early diagnosis of lung cancer is computed tomographic (CT) scan, which could reduce 20% mortality compared with chest X-ray; however, high false-positive together with radiation injury and high cost limited the widely use of CT in early diagnosis of lung cancer [19]. Circulating biomarkers were used to aid diagnosis, evaluate effectiveness of treatments, monitor recurrence after therapy, and predict prognosis [20]. A variety of serologic markers were now used in lung cancer diagnosis and prognosis, including carcinoembryonic antigen (CEA) [21], carbohydrate antigen 19-9 (CA 19-9) [22], cancer antigen 12-5 (CA125) [23], neuron-specific enolase (NSE), and cytokeratin 19 fragments (Cyfra21-1) [24]; however, most of these markers showed better sensitivity only in advanced stages lung cancer patients (III + IV) and had limited value in early lung cancer diagnosis [25].

In this study, we showed that level of circulating HSP70 significantly decreased in lung cancer patients compared with healthy controls (*P* < 0.0001). Receiver operating characteristic (ROC) analysis showed that HSP70 (AUC: 82.2%, SN: 74.1%, SP: 80.0%) had higher diagnosis value than clinical existing biomarkers CEA (AUC: 80.1%, SN: 76.8%, SP: 67.3%) and CA 19-9 (AUC: 63.7%, SN: 64.2%, SP: 54.0%). In early lung cancer patients' analysis, HSP70 was found to have more diagnosis valuable (AUC: 83.8%, SN: 71.2%, SP: 84.0%) than CEA (AUC: 73.7%, SN: 73.2%, SP: 69.1%) and CA 19-9 (AUC: 61.5%; SN: 69.4%; SP: 53.4%). In addition, HSP70 showed more valuable in the diagnosis of SCC (AUC: 85.9%, SN: 86.1.9%, SP: 81.0%) than ADC (AUC: 81.0%, SN: 69.1%, SP: 81.0%). Combined analysis of HSP70 and existing biomarker: CEA and CA 19-9 exhibited that HSP70 combined CEA and CA 19-9 showed the highest AUC (0.945, 95% CI, 0.855–1.000). All these resulted indicated that HSP70 was important in early lung cancer diagnosis.

HSP70 is frequently found on the plasma membrane of a large number of malignant tumors including nonsmall cell lung cancer (NSCLC) and gets released into the blood circulation in lipid vesicles [26]. Furthermore, differential expression of HSP70 is associated with the malignant phenotype of NSCLC cell lines and plays an important regulatory role in NSCLC cell proliferation [27]. Research has also shown that the expression of HSP70 increased in breast cancer cells and high expression of HSP70 in ovarian cancer with short survival period; therefore, HSP70 can be used as one of the effective indicators of ovarian cancer prognosis assessment [28]. HSP enhanced the tumorigenesis by regulating apoptotic pathways, for example, HSP70 could block Bax translocation to the mitochondria and prevent the release of cytochrome c; prevent recruitment of caspase-9 to the apoptosome by binding Apaf-1 [29]. HSP70 also supported tumor growth by inhibiting senescence. All these studies confirmed the importance of HSP70 in tumor growth, diagnosis, and prognosis [30]. However, the shortcoming of these studies was that there lacked of diagnosis study in lung cancer, and the enrolled patients were less than 100 [6, 8, 9, 13].

Our study first confirmed the role of circulating HSP70 in lung cancer diagnosis by detection of lung cancer plasmas and combination analysis of HSP70 and CEA and CA 19-9. The levels of HSP70 in lung cancer plasma were dramatically decreased, which is contrary to former studies that HSP70 was upregulated in tumor patients [6]. The reason for this paradoxical result was that our study detected circulating HSP70 in plasma, while other studies measured HSP70 in lipid vesicles, but further research should be performed to confirm our hypothesis. The most important development was that we established the AUCs for biomarker panels and determined the appropriate balance between sensitivity and specificity in the choice of cutoff point. In addition, we identified that the efficiency of HSP70 in combination with CEA and CA 19-9 was superior to that of the individual biomarkers alone for the discrimination of NSCLCs from healthy controls; new variable models for NSCLCs were created on the basis of equations obtained by binary logistic regression. The results showed that the efficiency of HSP70 in combination with CEA and CA 19-9 was superior to the individual biomarkers alone for the diagnosis of NSCLCs from healthy controls. The second important observation was that circulating HSP70 were dramatically decreased in older SCC patients (>65 years compared with <45 years, *p* < 0.05). In addition, the plasma HSP70 levels in SCC patients have prominently increased in advanced lung cancer patients compared to early stage SCC patients (*P* < 0.05). The lower HSP70 levels were also found in SCC patients with no metastasis compared to metastasis (*P* < 0.05). The results of this analysis indicated the importance of HSP70 in SCC patients.

The major limitation of our study was that all enrolled patients were diagnosed in the past years and no prognostic information available, so we were unable to identify the role of circulating HSP70 in poor prognosis of lung cancer. Another limitation of the study was a small size of CEA (34 patients) and CA 19-9 (19 patients) which might bias the results of the combination analysis.

In summary, our study identified that the combination of decreased HSP70, elevated CEA and CA19-9, could be used in lung cancer diagnosis, especially patients in early stage (stage I and II). Further researches should be performed to enlarge populations (lung cancer patients and healthy controls) in detection to draw a more impartial conclusion about the circulating HSP70 in lung cancer diagnosis; we also need to follow-up the enrolled patients to confirm the role of circulating HSP70 in predicting prognosis of lung cancer.

## 5. Conclusion

Decreased plasma HSP70 level combined with elevated classical circulating biomarkers CEA and CA 19-9, correlated closely with diagnosis of lung cancer, and combination of HSP70, CEA, and CA19-9 also contributed to early (stage I and II) diagnosis of lung cancer.

## Figures and Tables

**Table 1:** The characteristics of the lung cancer patients and healthy controls.

Characteristic	Lung cancer patients (197)	Healthy controls (100)
*Sex*		
Male	121	28
Female	76	72
*Age (years)*		
<45	23	56
45–65	124	35
>65	50	9
*Histology*		
Adenocarcinoma	126	
Squamous cell	43	
Others	28	
*Stage*		
Early	118	
Advanced	60	
Unknown	19	
*Metastasis*		
Yes	48	
No	148	
Unknown	1	
*Differentiation*		
Low	55	
Moderate	50	
High	12	
Unknown	80	
*Smoking*		
Yes	92	
No	92	
Unknown	13	
*Tumor size*		
≥3 cm	97	
<3 cm	70	
Unknown	30	

**Table 2:** Summary of SN, SP, and AUC for HSP70, CEA, and CA 19-9.

	HSP70				CEA	CA 19-9
	All	ADC	SCC	Early		
SN (%)	74.1	69.1	86.1	71.2	83.9	84.2
SP (%)	80.0	81.0	81.0	84.0	74.5	50.0
AUC (%)	82.2	81.0	85.9	83.8	82.0	69.5

**Table 3:** The diagnostic efficiency of model in NSCLC and controls.

	AUC (95% CI)	SN (%)	SP (%)	Positive LR	Negative LR
NSCLC versus control					
HSP70 + CEA + CA 19-9	0.945 (0.855–1.000)	75.0	100		0.25
HSP70	0.898 (0.764–1.000)	75.0	93.8	12.1	0.27
CEA + CA 19-9	0.844 (0.687–1.000)	100	62.5	2.7	0
CEA	0.836 (0.672–1.000)	87.5	81.3	4.7	0.28
CA 19-9	0.773 (0.565–0.982)	75.0	68.8	2.4	0.36

## Data Availability

The data used to support the findings of this study are available from the corresponding author upon request.
